# Foreign Bodies in Bronchial Tubes

**Published:** 1889-08-17

**Authors:** 


### Within the Wards.

### FOREIGN BODIES IN BRONCHIAL TUBES.

When hard substances are swallowed, it is well known that they occasionally pass down the windpipe instead of down the gullet into the stomach. Usually, a foreign body in the windpipe, or rather in a bronchial tube, gives rise to very decided symptoms, such as difficult breathing or violent coughing efforts at expulsion. But this is not always the case; and, as it is always most desirable to have the foreign body removed at once, whether it give rise to urgent symptoms or not, the following cases, recorded by Mr. Thomas Bryant, F.R.C.S., surgeon to Guy's Hospital, are well deserving of the consideration of the general reader. The first case was that of a man forty-nine years of age, who fell fast asleep whilst sucking a button which he had put into his mouth. He awoke with a gasp, and, finding that the button had disappeared, tried to bring it up by coughing. Not succeeding in that, he took an emetic; but this also failing, he betook himself to Guy's Hospital. He was admitted and examined; but, as he had suffered from bronchitis, it was difficult to say with certainty that the button was actually in the chest. He himself thought he felt it on the left side. An operation was suggested to him, which would have at once cleared up the doubt and got rid of the button if it had been found. The man, like many other foolish persons, declined the operation, and took upon himself the risk of losing his life. He remained in the hospital twenty-six days, with the cough still continuing, and then went out. Some weeks later he went to St. Saviour's Infirmary and was put under the care of Dr. Gross. He then looked thin and ill, had a constant cough, and expectorated large quantities of very offensive matter. In ten days from his admission he died of exhaustion. After death the button was found in one of the bronchial tubes in such a position that its removal would have been comparatively easy. The man by refusing an operation, which could have been performed under an anæsthetic, causing no pain, undoubtedly lost his life.

The second case was that of a little boy seven years old, who whilst playing with a trumpet unluckily swallowed, or rather inhaled, the mouthpiece. He was taken to Guy's within three hours of the accident and carefully examined. The chief symptom, confirmatory of the story, was a momentary cough when the mouthpiece first disappeared, and a persistent whistling sound every time the patient took a long breath with his mouth closed. Otherwise the evidences of a foreign body in the chest were very uncertain. But as the symptoms did not abate on treatment, an operation was performed thirteen days after the accident. The windpipe was opened, and a pair of forceps pushed down five and a half inches in the direction of the bronchial tubes. The offending mouthpiece was promptly caught and removed, with the gratifying result of a rapid and complete recovery. It is well for everbody to know that tracheotomy in a healthy person is neither a painful nor a dangerous operation. If there be a suspicion amounting to reasonable certainty that any hard substance has been taken into the air passages, the wise thing to do, without any hesitation, is to submit to an operation at the earliest moment when the surgeon thinks it ought to be done. Danger and death lie in the path of him who fears to trust himself to a judgment better than his own.

